# Outcome of training of maternal and child health workers in Ifo Local Government Area, Ogun State, Nigeria, on common childhood blinding diseases: a pre-test, post-test, one-group quasi-experimental study

**DOI:** 10.1186/s12913-019-4272-1

**Published:** 2019-06-27

**Authors:** A. O. Olowoyeye, K. O. Musa, O. T. Aribaba

**Affiliations:** 1 Me Cure Healthcare, Oshodi Lagos, Nigeria; 2 Department of Ophthalmology (Guinness Eye Center), Lagos University Teaching Hospital/ College of Medicine of the University of Lagos, Idi-Araba, Lagos, Nigeria

**Keywords:** Training, Maternal and child health workers, Childhood blindness

## Abstract

**Background:**

Maternal and child health workers (MCHWs) are often the first point of contact with pregnant women, children, and caregivers. Therefore, they can play a significant role in early detection of causes of childhood blindness, facilitate prompt referral to specialized centers and provide health education to caregivers for preventive eye care.

**Methods:**

This is a pre-test, post-test, single group, quasi-experimental study to evaluate the outcome of training MCHWs on common blinding childhood diseases. All MCHWs in Ifo Local Government Area were selected to participate in the study. Pre-training, qualitative data was obtained from two focus group discussions while quantitative data was obtained using a self-administered questionnaire. Three months post-training, quantitative data was obtained using the same self-administered questionnaire as was used pre-training. Total and percentage scores on the pre- and post-tests were calculated for each participant. A score of ≥70% was regarded as sufficient while < 70% score was regarded as insufficient. McNemar’s test was used to determine differences in proportions between pre- and post-training quantitative measurements.

**Results:**

Of the 65 MCHWs in the Local Government Area, 61 participated in the study giving a response rate of 93.8%. The age range of study participants was from 28 to 57 years with a mean age of 41 ± 8.3 years. The male: female ratio was 1:7.7. During the focus group discussions, measles was the most commonly mentioned cause of childhood blindness however, participants showed more knowledge of the signs and symptoms of new-born conjunctivitis. Based on a sufficient knowledge score of ≥70%, only one participant (1.6%) demonstrated sufficient knowledge on quantitative survey pre-training. Post-training, there was a statistically significant increase (20, 32.8%) in the proportion of participants with sufficient knowledge (McNemar’s test *p* = .000).

**Conclusions:**

This study demonstrated that the training of MCHWs on common childhood blinding diseases (such as congenital cataract and congenital glaucoma) had the potential to improve knowledge regarding prevention, prompt recognition and early referral of common treatable potentially blinding diseases.

## Background

In most developing countries, there is a concentration of medical personnel in urban areas to the neglect of rural areas where most of the children in need of health care reside [1]. Primary Health Care (PHC) aims to address this issue by bringing health care closer to those in need [2]. These services appear to be making a difference as simple interventions at the primary care level like Vitamin A supplementation and measles immunization have been suggested as reasons for the decline in corneal blindness among children [3]. Maternal and child health workers (MCHWs) have been central to this improvement; they should be motivated and empowered to intensify efforts to achieve complete eradication of avoidable corneal blindness. Nigeria is a low-middle income country in West Africa with an extrapolated prevalence of childhood blindness of 0.8 per 1000 children [4]. With the decline in corneal blindness in Nigeria, cataract is emerging as the commonest avoidable cause of childhood blindness [3]. Studies have shown that even where highly specialized facilities for management of pediatric cataract exist, late presentation with poor visual outcome is a significant cause of ocular morbidity [5]. This is because there is a level of urgency in treating childhood cataracts to prevent irreversible amblyopia [6]. MCHWs can play a significant role in early detection of childhood cataract if they have the knowledge to recognize and promptly refer [7].

PHC centers are relatively uniformly distributed throughout Nigeria [8], therefore, through outreaches and health promotion campaigns, preventive eye care can be offered to children with limited ability to access prompt treatment. For MCHWs to be effective in these, they must have adequate knowledge otherwise information passed on to caregivers may prove detrimental [9–11].

This study is one of very few studies in West Africa to determine if MCHWs have good knowledge of the common causes of childhood blindness. It also determined the effect of training MCHWs on health education, prevention, and recognition and referral of blinding childhood diseases. This information may be used to advocate for special training and re-training of MCHWs on prevention as well as early detection and prompt referral of leading causes of childhood blindness.

## Methods

This was a pre-test, post-test, single group quasi-experimental study. The study took place over a 3-month period (August–November 2016) in Ifo Local Government Area of Ogun State, Nigeria. The study population included primary healthcare workers who give direct health care to pregnant women and children in PHC facilities in Ifo Local Government Area. These are healthcare workers within the following cadres: Nurses/Midwives, Community Health Officers (CHOs) and Community Health Extension workers (CHEWs).

Ethical approval was obtained from the Health Research Ethics Committee of the Lagos University Teaching Hospital (Protocol number: ADM/DCST/HREC/APP/1061; approval dates 08-07-2016 to 08-07-2017.) The study adhered strictly to the Helsinki declaration. Approval was obtained from Ifo Local Government Area Medical Administrator and written informed consent was obtained from all study participants.

Purposive sampling was used to select two Maternal and Child Health Workers (MCHWs) [One nurse and one CHEW/CHO] per ward to participate in the focus group discussions. Nurses and CHEWs/CHOs from 10 wards balloted separately for one slot each. The 11th ward had primary health facilities manned by CHEWs only; one CHEW was selected from that ward by balloting.

For the questionnaire survey, to achieve sufficient knowledge of common blinding eye conditions in children in 80% of MCHWs after training, a minimum sample size of 294 was calculated using the formula for comparison between two groups when endpoint is a qualitative variable [12]. The standard normal deviate was set at 1.96; the proportion of PHC workers with good knowledge of common eye diseases in a previous study was taken as 68.7% [13] while the desired level of precision was set at 5%. Considering this study assesses a finite population [14], the sample size was adjusted for the Finite Population Correction [15]. This yielded a minimum sample size of 53 which was further increased to 59 after allowance for 10% attrition. To further increase the power of the study, all willing and consenting MCHWs in Ifo Local Government Area that met the inclusion criteria were enrolled in the study. Pre-survey activities commenced with visits by the lead investigator to selected PHC facilities in Ifo Local Government Area. Following this, the study instrument (Pre-test and post-test questionnaires) and training curriculum were developed. Team members were then trained after which a pilot study to validate the study instrument was conducted at a PHC in a Local Government Area in the neighboring state (Lagos State)**.**

The pre-test, focus group discussions and training on childhood blindness were made to coincide with the monthly meeting at the Local Government Headquarters between the Medical Administrator and PHC workers. This provided the opportunity to access the entire MCHWs in the LGA at the same time. A pre-test was conducted on the 4th of August 2016. The pre-test was in the form of a self-administered questionnaire. The pre-test took place in the conference room at the Local Government headquarters. It was undertaken at the same time by all the study participants in the presence of the lead researcher and two research assistants. In the first section of the pre-test, study participants were asked to identify congenital cataract, congenital glaucoma and newborn conjunctivitis from colored pictures. The same eye conditions were presented in the pre-test and post-test however the pictures were different. The second section had 11 multiple choice questions which were the same in the pre- as well as the post-test. These assessed participants’ knowledge of the leading causes of childhood blindness (Measles, Vitamin A deficiency, eye injury, congenital cataract congenital glaucoma, and newborn conjunctivitis) as well as their signs and symptoms, treatment and prevention. In the third section participants were asked to choose between immediate referral and treatment for eye conditions assessed in the second part of the test. The last section of the questionnaire assessed barriers to child eye care. This was followed by two focus group discussions. The first focus group discussion comprised nurses only; the second focus group discussion took place immediately after the first and was comprised of CHEWs only. The segregation was done to ensure that discussants freely express themselves. The focus group discussions were conducted in English and they explored knowledge of the leading causes of childhood blindness, how these are identified, managed and prevented. A research assistant recorded the proceedings with a smartphone camera and a tape recorder. The lead investigator also took notes during the discussions. A training session was then provided (the curriculum for the training was developed by the authors using a modified version of the Prevention of childhood blindness teaching set [16] and also based on the Nigerian standing orders for CHOs and CHEWs [17]. The training consisted of a power point presentation followed by a practical session. After the training, the MCHWs were advised to keep a record of children with eye diseases that they see during the study period and referrals were to be made to a designated eye center within the local government. The lead investigator was to be informed of all referrals and her contact information was to be given to the referred children’s caregivers. These referrals were included in the data analyzed by the principal researcher. Educational materials in the form of booklets with pictures depicting common blinding childhood eye diseases to educate caregivers were given to the MCHWs. A post-test using the same self-administered questionnaire from the pre-test was administered 3 months after the pre-training on November 10th^,^ 2016.

### Data analysis

Data was entered into IBM-SPSS version 23 (© Copyright IBM Corporation 2014 Armonk NY). Total and percentage scores on the pre- and post-tests were calculated for each participant. A score of ≥70% was regarded as sufficient while < 70% score was regarded as insufficient. The authors chose 70% as the threshold for “sufficient (Good)” and “insufficient (Poor”) scores because it is expected that the study participants should have average knowledge having undergone basic healthcare training whose curriculum is expected to have covered the common causes of childhood blindness. Further training should therefore aspire to impart above average knowledge with expectations of excellent scores ≥70%. McNemar’s test for two paired proportions was used to specifically examine changes pre- and post-training. Level of statistical significance was set at 5%.

Data from the focus group discussions was transcribed from the audiovisual devices and comments entered into Microsoft Excel Version 3, 2016. Comments were arranged for each interview question such that answers from the two focus group discussions were together. Major themes were identified and illustrative quotes were noted.

## Results

### Characteristics of focus group discussions participants

Twenty-one MCHWs participated in the two focus group discussions. One group was made up of 10 female nurses while the other group had 10 females and 1 male CHEW. The ages ranged from 30 to 57 years with a mean age of 43 ± 8.8 years. All the focus group discussions participants indicated that they had eye care training during their basic healthcare training while 2 (9.5%) of them also had post-basic eye care training. Post-basic eye care training is eye care training obtained after graduation from basic healthcare training. This includes graduate and postgraduate training (BSC, Masters, PHD),as well as certificate courses obtained from on-the-job, classroom or online training courses offered by accredited training institutions.

### Summary of findings from the focus group discussions

1. Of the leading causes of childhood blindness, the most commonly mentioned was measles. Other conditions mentioned include new-born conjunctivitis, eye injury and harmful traditional practices. The most common harmful traditional practices mentioned were instillation of the following substances into the eye: breast milk, cow and human urine.

#### Illustrative quotations from the focus groups

“Measles causes blindness in the two eyes in a lot of children but I have also seen a child that went blind in one eye after a teacher flogged her” Female Nurse

2. New-born conjunctivitis was the most clearly elucidated eye disease. Discharge and sticking together of the eyes are symptoms well known to participants. Red eye was the commonest ocular symptom associated with measles.

#### Illustrative quotations from the focus groups.

“We see babies with discharge and gumming together of the eyes after they are born. We know it is new-born conjunctivitis and we know it is because their mothers have vaginal infection; that is why they have the condition.” Female Nurse

3. While some participants treat new-born conjunctivitis, others refer immediately to the nearest General Hospital. For the management of measles, all participants mentioned vitamin A supplementation as part of the treatment protocol in their routine practice. Most participants refer children with eye injury however some treat only referring if there is history of vision loss.

#### Illustrative quotations from the focus groups.

“We advise mothers to clean the eyes of new-born babies that have eye discharge with warm water with salt sprinkled in it and then apply Gentamicin eyedrop” Female CHEW

4. On prevention and health promotion, participants mentioned that during health talks given during immunization clinics, the importance of breastfeeding is emphasized. Vitamin A supplementation of mothers and children was mentioned as routine practice by all participants. However, the importance of vitamin A in the diet and local sources of vitamin A were not specifically mentioned. Measles immunization was specifically mentioned by all participants. Only one participant was aware that Rubella vaccination plays a role in preventing childhood blindness. Participants agreed that counselling and treating mothers with Sexually Transmitted Diseases is a method of new-born conjunctivitis prevention. Prophylactic antibiotic/antiseptic instillation into babies’ eyes at birth to prevent new-born conjunctivitis was not routine practice among study participants. The reason given for this is the unavailability of required drugs.

#### Illustrative quotations from the focus groups.

“We tell pregnant women that if they have vaginal discharge or itching they should let us know so we can treat it before the child is born. That way, the child’s eyes will not be infected when he/she is born” Female CHEW

5. Participants did not routinely ask caregivers about concerns regarding children’s eyes or attempt to examine children’s eyes after birth or during immunization visits.

#### Illustrative quotation from the focus groups.

“We check if a child can hear after he/she is born but children do not open their eyes right after birth and they do not really see so there is no point checking the eyes if the mother has concern, we expect her to come and tell us later.” Female nurse.

“We don’t really have time for checking children’s eyes as we are short staffed. It is one person that performs immunization, weighing and recording; this takes time so we cannot be checking eyes as well.” Female Nurse.

### Questionnaire survey

#### Characteristics of questionnaire survey participants

Of the 65 MCHWs in the Local Government Area, 61 participated in the questionnaire survey giving a response rate of 93.8%. The age range was from 28 to 57 years with a mean age of 41 ± 8.3 years. The male: female ratio was 1:7.7. The sex and age distribution of the participants is summarized in Table 1. Thirty-nine (63.9%) of the participants were CHEWs. Forty (65.6%) participants had at least 10 years’ experience (Table 2). All 61 study participants indicated that they had eye care training during their basic healthcare training while 8 (13.1%) participants had post-basic eye care training.

**Table 1:** Socio-demographic characteristics of participants

	Questionnaire participants		Focus group participants	
	Frequency %		Frequency %	
Age (years)				
20–29	5	8.2	0	0.0
30–39	22	36.1	7	33.3
40–49	19	31.1	8	38.1
≥50	12	19.7	6	28.6
Age not indicated	3	4.9	0	0.0
Total	61	100.0	21	100.0
Gender				
Male	7	11.5	1	4.8
Female	54	88.5	20	95.2
Total	61	100.0	21	100.0

**Table 2:** Professional profile of participants

	Questionnaire participants		Focus group participants	
	Frequency%		Frequency%	
Designation				
Nurse	19	31.2	10	47.6
Community Health Officer	3	4.9	0	0.0
Community Health Extension worker	39	63.9	11	52.4
Total	61	100.0	21	100.0
Qualification				
University degree	5	8.2	3	14.3
Public health nursing certificate	2	3.3	1	4.7
Registered nurse/midwife certificate	13	21.3	6	28.6
Community Health officer certificate	26	42.6	6	28.6
Community Health technician certificate	15	24.6	5	23.8
Total	61	100.0	21	100.0
Years of experience				
1–4	6	9.8	1	4.8
5–9	15	24.6	4	19.0
10–14	19	31.1	8	38.1
15–19	9	14.8	3	14.3
≥20	12	19.7	5	23.8
Total	61	100.0	21	100.0

Based on the grading of knowledge into sufficient (percentage score ≥ 70%) and insufficient (percentage score < 70%), only one participant (1.6%) demonstrated sufficient knowledge on quantitative survey pre-training. Post-training, there was a statistically significant increase (20; 32.8%) in the proportion of participants with sufficient knowledge (McNemar’s Test *p* = .000).

Statistically significant increases were found between pre- and post-training survey in knowledge of some of the common causes of childhood blindness (Table 3); picture identification of the cataract, congenital glaucoma and new-born conjunctivitis (Table 4); knowledge of some locally available sources of vitamin A (Table 5); and some of the methods of preventing new-born conjunctivitis (Table 6). There was no statistically significant difference between pre- and post-test knowledge of immunizations that prevent childhood blindness (Table 7) as well as knowledge of definitive treatment of glaucoma or cataract (Table 8). Pre-training, immediate referral was the most commonly selected management option for cataract, red eye and newborn conjunctivitis. Post-training, there was a statistically significant increase in the proportion of study participants that would immediately refer children with cataract (Table 9).

**Table 3:** Changes in responses on common causes of childhood blinding diseases

Disease					
Measles	Pre-training		Post-training		*p-value 0.006^ǂ^
			Correct	Incorrect	
		Correct	49	1	
		Incorrect	11	0	
Eye injury	Pre-training		Post-training		*p-value 0.345
			Correct	Incorrect	
		Correct	30	11	
		Incorrect	17	3	
Newborn conjunctivitis	Pre-training		Post-training		*p-value 0.345
			Correct	Incorrect	
		Correct	27	11	
		Incorrect	17	6	
Vitamin A deficiency	Pre-training		Post-training		*p-value .036^ǂ^
			Correct	Incorrect	
		Correct	29	8	
		Incorrect	20	4	
Cataract	Pre-training		Post-training		*p-value 0.031^ǂ^
			Correct	Incorrect	
		Correct	26	9	
		Incorrect	22	4	
Glaucoma	Pre-training		Post-training		*p-value 0.001^ǂ^
			Correct	Incorrect	
		Correct	27	5	
		Incorrect	23	6	

*McNemar’s test

^ǂ^Statistically significant

**Table 4:** Changes in responses on picture identification of common childhood blinding diseases

Disease					
Cataract	Pre-training		Post-training		*p-value 0.000^ǂ^
			Correct	Incorrect	
		Correct	25	3	
		Incorrect	32	1	
Newborn conjunctivitis	Pre-training		Post-training		*p-value 0.000^ǂ^
			Correct	Incorrect	
		Correct	19	1	
		Incorrect	38	3	
Congenital glaucoma	Pre-training		Post-training		*p-value 0.000^ǂ^
			Correct	Incorrect	
		Correct	1	0	
		Incorrect	50	10	

*McNemar’s test

^ǂ^ Statistically significant

**Table 5:** Changes in responses on knowledge of locally available sources of Vitamin A

Locally available sources of vitamin A					
Palm oil	Pre-training		Post-training		*p-value 0.143
			Correct	Incorrect	
		Correct	37	7	
		Incorrect	15	2	
Breast milk	Pre-training		Post-training		*p-value 0.029^ǂ^
			Correct	Incorrect	
		Correct	27	9	
		Incorrect	22	3	
Green leafy vegetables	Pre-training		Post-training		*p-value 0.004^ǂ^
			Correct	Incorrect	
		Correct	17	9	
		Incorrect	27	8	
Mango	Pre-training		Post-training		*p-value 0.000^ǂ^
			Correct	Incorrect	
		Correct	15	4	
		Incorrect	31	11	
Beans	Pre-training		Post-training		*p-value 0.382
			Correct	Incorrect	
		Correct	2	12	
		Incorrect	10	37	

*McNemar’s test

^ǂ^ Statistically significant

**Table 6:** Changes in responses on methods of prophylaxis for newborn conjunctivitis

Mode of newborn conjunctivitis prophylaxis					
Prevent/treat maternal STIs	Pre-training		Post-training		*p-value 0.243
			Correct	Incorrect	
		Correct	23	14	
		Incorrect	22	2	
Clean newborn eyes at birth	Pre-training		Post-training		*p-value 0.000^ǂ^
			Correct	Incorrect	
		Correct	15	9	
		Incorrect	33	4	
Conjunctival instillation of tetracycline at birth	Pre-training		Post-training		*p-value 0.118
			Correct	Incorrect	
		Correct	4	4	
		Incorrect	11	42	
Conjunctival instillation of povidone iodine at birth	Pre-training		Post-training		*p-value 0.041^ǂ^
			Correct	Incorrect	
		Correct	2	5	
		Incorrect	15	39	
Conjunctival instillation of silver nitrate at birth	Pre-training		Post-training		*p-value 0.021^ǂ^
			Correct	Incorrect	
		Correct	0	3	
		Incorrect	13	45	

*McNemar’s Test

^ǂ^ Statistically significant

**Table 7:** Changes in responses on immunizations that prevent blinding eye diseases

Measles immunization			Post-training		*p-value 1.000
			Correct	Incorrect	
	Pre-training	Correct	28	14	
		Incorrect	13	6	
Rubella immunization			Post-training		*p-value 0.078
			Correct	Incorrect	
	Pre-training	Correct	6	6	
		Incorrect	15	34	

*McNemar’s Test

**Table 8:** Changes in responses on definitive treatment of cataract and glaucoma

Definitive treatment of cataract			Post-training		*p-value 0.185
			Correct	Incorrect	
	Pre-training	Correct	19	10	
		Incorrect	18	14	
Definitive treatment of Glaucoma			Post-training		*p-value 0.362
			Correct	Incorrect	
	Pre-training	Correct	2	12	
		Incorrect	18	29	

*McNemar’s Test

**Table 9:** Changes in responses on practices regarding childhood blinding diseases

		Post-training			*p-value 0.031^ǂ^
			Immediate referral	Treat first	
Practices regarding cataract	Pre-training	Immediate referral	40	4	
		Treat first	14	3	
		Post-training			*p-value 0.087
			Immediate referral	Treat first	
Practices regarding red eye(s) in a child	Pre-training	Immediate referral	26	9	
		Treat first	19	7	
		Post-training			*p-value 0.711
			Immediate referral	Treat first	
Practices regarding newborn conjunctivitis	Pre-training	Immediate referral	27	16	
		Treat first	13	5	

*McNemar’s Test

^ǂ^Statistically significant

There was a statistically significant increase in the proportion of study participants that indicated that they had given health talks with child eye care related content in the preceding three months between pre- and post-training survey (McNemar’s Test *p* = 0.000).

Inadequate training was identified by 28 (45.9%) participants as the reason why they were incapable of child eye care. Other reasons given are shown in Table 10. With training, there was a statistically significant increase in the number of study participants (29; 47.5%) that indicated that they were capable of child eye care (McNemar’s Test *p* = .031).

**Table 10:** Barriers to child eye care pre- and post-training

Reasons	Pre-training		Post-training	
	Frequency	%	Frequency	%
Inadequate training	28	45.9	22	36.1
Inadequate time	5	8.2	0	0.0
Inadequate supervision	3	4.9	3	4.9
Unavailability of drugs/equipment	12	19.7	9	14.8
Other	3	4.9	9	14.8

### Referrals from study participants during study period

The lead investigator was directly informed of six children being referred for ocular complaints through phone calls from MCHWs that participated in the survey. Three of the referred children were seen. This included a 3-year-old girl with squint, an 8-month-old boy with retinoblastoma and a 4-year-old boy with vernal conjunctivitis. The remaining three referred children were not seen at all.

## Discussion

All the MCHWs that participated in this study had previously had eye care training as part of their basic healthcare training which is consistent with the training curricular of PHC workers [18, 19]. However, only one study participant demonstrated sufficient knowledge about leading causes of childhood blindness pre-training. This may be because over 60% of the study participants had basic eye care training more than 10 years prior to the study. Also, of those that had retraining, 50% had completed re-training more than 5 years before the study period.

Despite the good knowledge of measles prevention, recognition and management demonstrated during the qualitative survey, less than 70% of the MCHWs picked measles immunization as a means of preventing blinding eye disease in children on quantitative survey pre- and post-training. There is a nationwide schedule for measles immunization and a protocol for management of measles infection as stipulated in the National Primary Healthcare Development Agency (NPHDA)‘s National Standing Orders for CHOs/CHEWs [17] and outlined on posters on the walls of all PHC facilities visited. This may suggest that MCHWs imbibe knowledge by rote learning and from routine practice of schedules and protocols without deeper understanding.

In this study, pre-training, MCHWs demonstrated knowledge of the diagnosis and management of new-born conjunctivitis as it was widely discussed during qualitative survey. However, most focus group discussion participants mentioned that they do not routinely practice antibiotic/antiseptic prophylaxis. The reason given for the poor practice was that the required drugs were not available. Part of the essential drug list for PHC includes povidone Iodine [20]. However, this drug was not available in any PHC in the study by Onapoya et al. [21] In Tanzania, lack of eyedrops was one of the reasons given for the low level of practice of Crede’s prophylaxis among Reproductive and Child Health workers [7]. In Kenya [22], where tetracycline is readily available in most of the health facilities, majority of the MCHWs routinely applied tetracycline eye ointment in newborn eyes.

Population based surveys suggest that 15–35% of childhood blindness in Sub-Saharan Africa is due to congenital or developmental cataract [23]. In Nigeria, cataract was the leading cause of blindness in a recent study in schools for the visually impaired [3]. In this study, cataract was not mentioned at all as a common cause of childhood blindness in this environment during qualitative survey. Also, quantitative survey pre-training showed that only about half of the participants knew that it is a common cause of childhood blindness. Although it was the most commonly identified childhood blinding eye condition from a set of pictures, the percentage of study participants that recognized cataract was low (45.9%). Although this is higher than was recorded among Reproductive and Child Health workers in Tanzania (16.6%) [7], it is lower than what was found among PHC workers in northern Nigeria (62.5%)[13, 88%) in Tanzania [10]. After the training, a statistically significant increase in the proportion of MCHWs that could identify cataract was recorded. A similar increase in the identification of cataract by trained Reproductive and Child Health workers was reported in Tanzania [7].

An increase in the proportion of MCHWs that knew that surgery is the definitive treatment of cataract was found post-training. In addition, there was an increase in the proportion of MCHWs that indicated that they would immediately refer children with cataract. This demonstrates that with training an increased number of MCHWs could correctly identify cataract which may facilitate early referral of children to specialised centres thereby improving visual outcome. Congenital Rubella Syndrome is a prominent and clinically important cause of childhood cataract in our environment and rubella vaccination is recommended as a preventive and control measure [24]. The MCHWs demonstrated poor knowledge of rubella vaccination as a means of preventing childhood blindness. In view of the importance of this vaccine in the prevention of ocular and systemic morbidity, there is a need to strongly emphasize the need for rubella vaccination in the training curricular of PHC workers.

Only 1 (1.64%) participant recognized a picture of congenital glaucoma pre-training. Poor identification of congenital glaucoma was also found in a study in Kenya [25] where only 13.33% of PHC workers recognized a picture of congenital glaucoma. After the training, there was a statistically significant increase in the proportion of study participants that could recognize congenital glaucoma. This study demonstrates that MCHWs could be trained to recognize signs and symptoms of congenital glaucoma hence facilitating prompt referral.

After training, the MCHWs in this study demonstrated an improvement in knowledge regarding prevention and management of important causes of childhood blindness. The decrease in the proportion of participants that indicated they were not capable of child eye care after training may be due to the fact that training boosts confidence to practice. Therefore, training may be a way of encouraging and empowering MCHWs to take an interest in looking at children’s eyes during routine care in order to detect ocular pathology that may be amenable to treatment. The number of MCHWs that gave eye care related health education to caregivers increased after the training. Considering the fact that this study has demonstrated an increase in knowledge among the MCHWs regarding prevention and management of important causes of childhood blindness, the quality of information MCHWs possess and therefore pass on to caregivers can be improved upon with training.

Lack of drugs was identified as a barrier to the practice of new-born conjunctivitis prophylaxis during the focus group discussions. Similar findings was demonstrated in Ethiopia [26]. Studies to identify barriers that may limit the ability of MCHWs to effectively carry out health education, prevention, identification of childhood blinding diseases and appropriate referral should be carried out as these could lead to lack of motivation to practice after adequate training. Despite direct referral with the phone number of the lead investigator given to caregivers, some of the referred children were not seen at the referral center. There is therefore need to conduct further studies to determine why referred children do not present at referral centers.

The pre-test, post-test methodology was used in this study because the entire population of MCHWs in Ifo Local Government Area at the time of the study was 65 which was just a little over the calculated sample size of 59. Reducing this number by formation of groups will reduce the sample size which will affect the power of the study. Secondly, since the MCHWs work within the same locale and meet regularly, the trained group may share what they learnt and course materials with the untrained group thereby giving rise to a procedural confound. Therefore, findings from this study may not be directly extrapolated to the general population. While the use of real patients with eye pathologies instead of pictures may have elicited better diagnostic skills, this would have necessitated allotting more time and funds.

## Conclusion

This study has demonstrated that the MCHWs had insufficient knowledge regarding common blinding eye diseases in children in their environment and that training had the potential to improve their knowledge. In view of this, it is recommended that revisions of the training curriculum be done to update trainees on common causes of childhood blindness in their environment. Also this group of health workers may benefit from periodic medical education programs to continuously update their knowledge on child eye care. Studies to determine the cost-effectiveness of the trainings could help policy makers understand their benefit. Furthermore, studies should be conducted to determine why some referred children are not seen at referral centers.

## Data Availability

The datasets generated and/or analyzed during the current study are available from the corresponding author on reasonable request.

## Abbreviations

CHEW: Community Health Extension Worker
CHO: Community Health Officer
MCHW: Maternal and Child Health worker
PHC: Primary Health Care
